# Use of saliva to monitor meningococcal vaccine responses: proposing a threshold in saliva as surrogate of protection

**DOI:** 10.1186/s12874-018-0650-3

**Published:** 2019-01-05

**Authors:** Mariëtte B. van Ravenhorst, Fiona R. M. van der Klis, Debbie M. van Rooijen, Elisabeth A. M. Sanders, Guy A. M. Berbers

**Affiliations:** 1 Centre for Infectious Disease Control, National Institute for Public Health and the Environment (RIVM), Postbaknummer 41, Postbus 1, 3720 BA Bilthoven, The Netherlands; 2 Department of Paediatric Immunology and Infectious Diseases, Wilhelmina Children’s Hospital, University Medical Center, Utrecht, The Netherlands

**Keywords:** *Neisseria meningitidis*, Correlate of protection, Salivary surrogate of protection, Threshold, Conjugate meningococcal vaccine

## Abstract

**Background:**

Mucosal antibodies against capsular polysaccharides offer protection against acquisition and carriage of encapsulated bacteria like *Neisseria meningitidis* serogroup C. Measurements of salivary antibodies as replacement for blood testing has important (cost-effective) advantages, particular in studies that assess the impact of large-scale vaccination or in populations in which blood sampling is difficult. This study aimed to estimate a threshold for meningococcal IgG salivary antibody levels to discriminate between unprotected and protected vaccinated individuals.

**Methods:**

MenA-, MenC-, MenW- and MenY-polysaccharide (PS) specific IgG levels in serum and saliva from participants in a meningococcal vaccination study were measured using the fluorescent-bead-based multiplex immunoassay. Functional antibody titers in serum against the four serogroups were measured with serum bactericidal assay using rabbit complement (rSBA). A threshold for salivary IgG was determined by analysis of ROC curves using a serum rSBA titer ≥128 as correlate of protection. The area under the curve (AUC) was calculated to quantify the accuracy of the salivary test and was considered adequate when ≥0.80. The optimal cut-off was considered adequate when salivary IgG cut-off levels provided specificity of ≥90%. True positive rate (sensitivity), positive predictive value, and negative predictive value were calculated to explore the possible use of salivary antibody levels as a surrogate of protection.

**Results:**

The best ROC curve (AUC of 0.95) was obtained for MenC, with an estimated minimum threshold of MenC-PS specific salivary IgG ≥3.54 ng/mL as surrogate of protection. An adequate AUC (> 0.80) was also observed for MenW and MenY with an estimated minimal threshold of 2.00 and 1.82 ng/mL, respectively. When applying these thresholds, all (100%) samples collected 1 month and 1 year after the (booster) meningococcal vaccination, that were defined as protective in the saliva test for MenC, MenW and MenY, corresponded with concomitant serum rSBA titer ≥128 for the respective meningococcal serogroups.

**Conclusion:**

The saliva test offers an alternative screening tool to monitor protective vaccine responses up to one year after meningococcal vaccination against MenC, MenW and MenY. Future (large) longitudinal vaccination studies evaluating also clinical protection against IMD or carriage acquisition are required to validate the currently proposed threshold in saliva.

## Background

Invasive meningococcal disease (IMD) remains a major public health concern due to the high mortality and morbidity. To prevent this invasive and potentially devastating infection, sufficient levels of functional antibodies in serum have been shown to be important [1]. Protective levels of antibodies in cases invasive infection are achieved only after several days [2], whereas the meningococcus can be fatal within hours after invasion in the bloodstream. Meningococcal polysaccharide (PS) conjugate vaccines (MCVs) are able to induce functional bactericidal serum antibodies that, upon binding to invading meningococci, activate complement leading to lysis of the bacteria and enhanced phagocytosis [3, 4]. Licensure of MCVs was based on serum bactericidal assay (SBA), rather than clinical efficacy studies [5]. The SBA measures antibody levels that induce complement mediated lysis of a specific target strain by incubation of two-fold serial dilutions of heat-inactivated serum with the targeted meningococcal strain in the presence of complement. A bactericidal titer of ≥8 is considered as correlate of protection baby rabbit complement [6, 7]. The use of baby rabbit complement may result in higher bactericidal titers than those obtained with human complement where a bactericidal titer of ≥4 is considered as correlate of protection [8]. Therefore, a more conservative threshold of ≥128 is often used in the SBA applying rabbit complement (rSBA) [1].

In the Netherlands, the annual incidence rate of IMD declined from 4.5 per 100.000 population in 2001 to 0.14 in 2014. This decline was mainly due to a gradual natural decline of the number of meningococcal serogroup B (MenB) cases [9], next to a rapid decline of meningococcal serogroup C (MenC) cases after introduction of the MenC conjugated vaccine in 2002 [10]. In 2002, a monovalent MenC conjugated to tetanus toxoid (MenC-TT) vaccine was offered to all children aged 1–18 years. At the same time, MenC-TT was introduced in the Dutch National Immunization Program (NIP) as a single vaccination for all children aged 14 months [10]. Historically, MenB was the most common serogroup in the Netherlands. However, meningococcal serogroup W (MenW) incidence is rising since the end of 2015 and currently MenW is the most common serogroup causing IMD in the Netherlands [11]. Meningococcal serogroup Y (MenY) disease is rare, and meningococcal serogroup A (MenA) IMD cases do not occur since 2004.

Saliva testing has previously been proposed as a potential method to monitor vaccine antibody and capsular polysaccharide antibody levels upon vaccination with conjugate vaccines, although the relationship between meningococcal salivary anticapsular antibodies and prevention of meningococcal carriage acquisition or protection against IMD is not defined [12–14]. Therefore, the relation of salivary antibodies with serum SBA titers needs to be evaluated. In addition, no previous studies aimed to determine a threshold of meningococcal specific antibodies in saliva for surrogate of protection against IMD.

In the present study, we compared MenA, MenC, MenW and MenY IgG levels in saliva with rSBA titers in serum of the corresponding serogroup in longitudinal samples taken before, one month and one year after vaccination with either the MenC-TT or tetanus toxoid conjugated quadrivalent MenACWY (MenACWY-TT) vaccine. Samples from a randomized trial aimed to determine non-inferiority of the MenACWY-TT vaccine as compared to the MenC-TT vaccine were used. With receiver operating characteristic (ROC) curve analysis of the SBA titers in serum (correlate of protection) and IgG levels in saliva (surrogate of protection), we estimated a threshold in saliva to discriminate between unprotected and protected vaccinated individuals.

## Methods

### Ethical statement

Ethical approval was obtained from the local Medical research Ethics Committees United (MEC-U). The trial was undertaken in accordance with the Good Clinical Practice guidelines established by the International Conference on Harmonization and with the Declaration of Helsinki. Written informed consent was obtained from both parents or guardians and subjects aged ≥12 years before enrollment. This study was registered at the EU Clinical Trials database (EudraCT number: 2013–001823-38) and at the Dutch Trial Register (www.trialregister.nl; NTR4430).

### Study population and design

Serum and saliva samples of a previous phase IV, single center, open-label controlled trial were used [15]. In short, 501 healthy participants of 10, 12 and 15 years of age received either the monovalent MenC-PS conjugated to tetanus toxoid (MenC-TT, NeisVacC©) or quadrivalent MenACWY-PS conjugated to tetanus toxoid (MenACWY-TT, Nimenrix©) vaccine at start of the study as a booster vaccination for MenC. All participants had been primed with a single dose of MenC-TT according to the Dutch Immunization Program at 14 months (age groups 10 and 12 years) and 3 years of age (group aged 15 years). Therefore, the antibody response for MenA, MenW and MenY reflects a primary response and for MenC a booster response. Blood and saliva samples were collected before, one month and one year after the study vaccination. Saliva samples were collected using the Oracol Saliva Collection system (Malvern Medical Developments Limited). Participants were instructed to allow absorption of saliva into the swab for 1 min. Saliva was immediately squeezed out the swab into a 2 mL spray-dried EDTA-tube (BD), and stored at ambient temperature. Within 24 h after collection, saliva samples were centrifuged and stored at -80̊C.

### Laboratory analyses

MenA-, MenC-, MenW- and MenY-PS specific IgG levels in serum and saliva were measured using the fluorescent-bead-based multiplex immunoassay (MIA) as previously described [12, 16, 17]. The functional antibodies in serum samples were assessed as previously described [18, 19].

### Statistical analyses

All statistical analyses were performed in the according to the protocol cohort as previously described [18]. For each serogroup, only samples with available SBA and salivary IgG results at all three time points were selected to prevent bias because antibody response after vaccination depends on antibody levels before vaccination. Participants with protective meningococcal antibodies at baseline developed higher immune responses after following the vaccination and showed better persistence of (protective) antibodies up to one year [15]. Serum and salivary antibody concentrations were log transformed before statistical analysis. Correlations were analyzed using Pearson correlation. A threshold for salivary IgG was determined by analysis with the ROC curve with an rSBA titer ≥128 used as correlate of protection. The area under the curve (AUC) was calculated to quantify the accuracy of the salivary test and was considered adequate when greater than 0.80 [20]. The optimal cut-off was considered adequate if the salivary IgG cut-off levels provided a specificity of ≥90%. To explore the possibility of the use of saliva antibody levels as surrogate of protection, true positive rate (TPR; sensitivity), positive predictive value (PPV), and negative predictive value (NPV) were calculated by classifying healthy participants with or without protective antibody levels in serum. For example, a PPV of 0.94 of the saliva test means that 94% of the healthy participants with a positive saliva teste (saliva antibody levels above the calculated cut-off) had protective rSBA titers (rSBA titer ≥128). Please note that PPV is not used in its normal diagnostic manner. A *p*-value below 0.05 was considered statistically significant. Data were analyzed using GraphPad Prism 7.00.

## Results

### Sample selection

For assessment of MenC antibody levels, longitudinal serum and saliva samples for all three time points were available from 416 participants, whereas these were available for MenA, MenW and MenY from 197, 200 and 199 participants, respectively. Overall, serum rSBA titers ≥128 were observed for 421/591 (71%), 863/1248 (69%), 419/600 (70%), and 458/597 (77%) samples for MenA, MenC, MenW and MenY, respectively. Table 1 shows the proportion of samples with an rSBA titer ≥128 for each time point and serogroup.

**Table 1:** The proportion of samples with an rSBA titer >=128 for meningococcal serogroup A (MenA), C (MenC), W (MenW) and Y (MenY) measured before (T0), one month (T1) and one year (T2) after the vaccination

Serogroup	T0 N (%)	T1 N (%)	T2 N (%)	All N (%)
MenA	32/197 (16)	195/197 (99)	194/197 (98)	421/597 (71)
MenC	34/416 (8)	415/416 (99)	414/416 (99)	414/416 (99)
MenW	22/200 (11)	198/200 (99)	199/200 (99)	419/600 (70)
MenY	60/199 (30)	199/199 (100)	199/199 (100)	458/597 (77)

### Correlation between serum serogroup-PS specific rSBA titers and IgG levels in serum and saliva

**Fig. 1:**
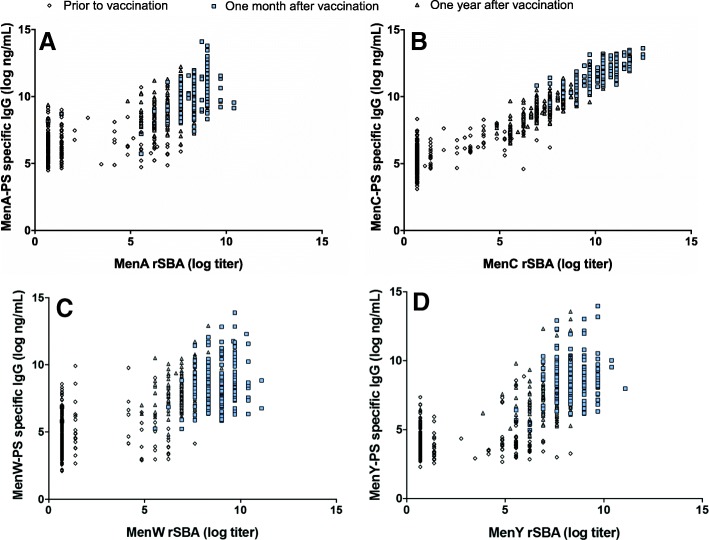
Correlation between polysaccharide (PS) specific IgG levels and functional antibody titers (SBA titers) in serum for meningococcal serogroup A (**a**), C (**b**), W (**c**), and Y (**d**)

Overall, serum antibody-PS specific IgG levels showed significant correlations with the corresponding rSBA titers for all four serogroups (Fig. 1a-d). The strongest correlation between serum rSBA titers and IgG levels in serum was observed for MenC after the booster vaccination (*R* = 0.96, *p*-value < 0.001; Fig. 1b) compared to the other serogroups after a single primary vaccination (*R* = 0.78, 0.74 and 0.76, for MenA, MenW and MenY, respectively, all *p*-values < 0.001; Fig. 1a, c-d). Correlations for each time point and serogroup are shown in Table 2.

**Table 2:** Correlation between serogroup-polysaccharide specific IgG levels and functional antibody titers (rSBA) in serum measured before (T0), one month (T1) and one year (T2) after the vaccination

Serogroup	T0 R (95% CI), *p*-value	T1 R (95% CI), *p*-value	T2 R (95% CI), *p*-value	All R (95% CI), *p*-value
MenA	0.11 (− 0.03–0.24), 0.14	0.34 (0.22–0.46), < 0.001	0.26 (0.13–0.39), 0.002	0.78 (0.75–0.81), < 0.001
MenC	0.64 (0.58–0.69), < 0.001	0.82 (0.78–0.85), < 0.001	0.84 (0.81–0.86), < 0.001	0.96 (0.96–0.97), < 0.001
MenW	0.01 (− 0.13–0.15), 0.85	0.23 (0.09–0.35), 0.0013	0.29 (0.16–0.41), < 0.001	0.74 (0.70–0.77), < 0.001
MenY	0.24 (0.11–0.38), 0.001	0.19 (0.05–0.32), 0.007	0.25 (0.12–0.38), 0.001	0.76 (0.72–0.79), < 0.001

Note. Abbreviations: *R* Pearson correlation coefficient, *CI* confidence interval

**Fig. 2:**
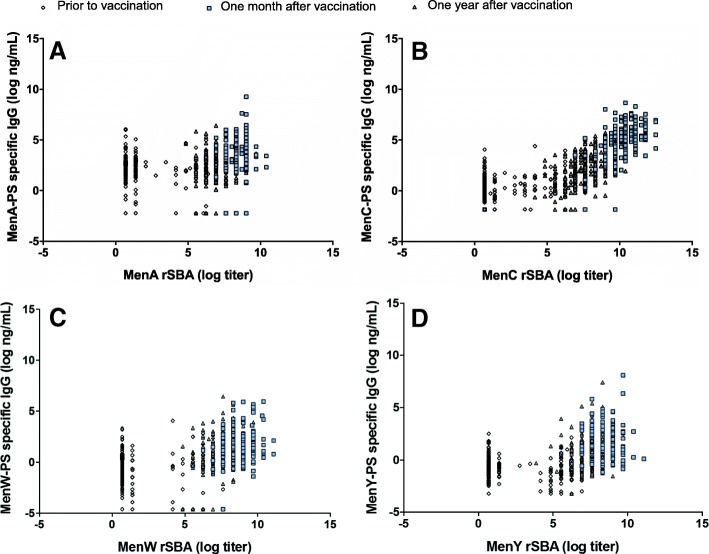
Correlation between polysaccharide (PS) specific IgG levels in saliva and functional antibody titers (SBA titers) in serum for meningococcal serogroup A (**a**), C (**b**), W (**c**), and Y (**d**)

MenC-PS specific IgG in saliva correlated with MenC rSBA titers in serum (*R* = 0.85, *p*-value < 0.001; Fig. 2b). MenA, MenW and MenY salivary antibody-PS specific IgG levels showed weak to modest correlation coefficients with the corresponding serum rSBA titers (*R* = 0.36, 0.60, 0.53, respectively, all *p*-values < 0.001; Fig. 2a, c-d). Correlations for each time point and serogroup are shown in Table 3.

**Table 3:** Correlation between sergroup-polysaccharide specific IgG levels and functional antibody titers (rSBA) in saliva measured before (T0), one month (T1) and one year (T2) after the vaccination

Serogroup	T0 R (95% CI), *p*-value	T1 R (95% CI), *p*-value	T2 R (95% CI), *p*-value	All R (95% CI), *p*-value
MenA	0.11 (− 0.03–.025), 0.11	0.11 (− 0.03–0.24), 0.13	0.06 (− 0.08–0.20), 0.42	0.36 (0.28–0.42), < 0.001
MenC	0.37 (0.29–0.45), < 0.001	0.54 (0.47–0.61), < 0.001	0.53 (0.46–0.60), < 0.001	0.85 (0.84–0.87), < 0.001
MenW	0.06 (− 0.08–0.20), 0.37	0.16 (0.02–0.29), 0.02	0.25 (0.11–0.37), 0.001	0.60 (0.54–0.65), < 0.001
MenY	0.02 (−0.12–0.15), 0.82	0.09 (− 0.05–0.23), 0.19	0.19 (0.05–0.32), 0.007	0.76 (0.72–0.79), < 0.001

Note. Abbreviations: *R* Pearson correlation coefficient, *CI* confidence interval

### Estimation of threshold in saliva as correlate of protection

**Fig. 3:**
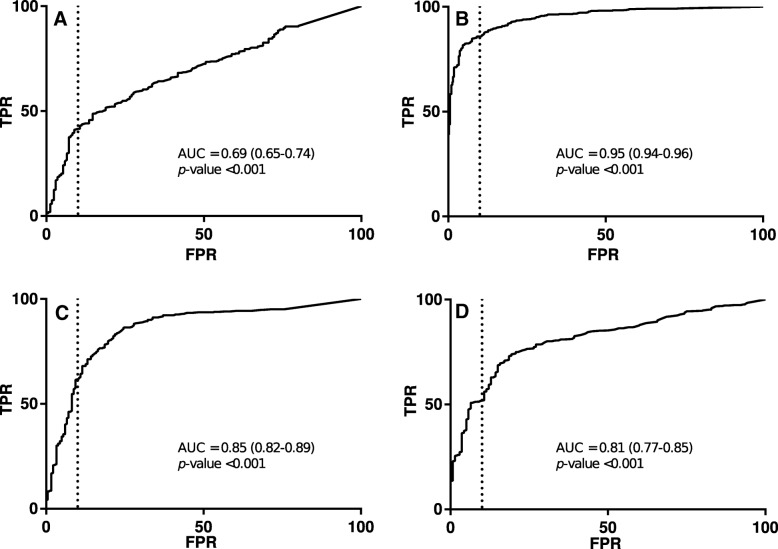
Receiver operating characteristic (ROC) curve analysis: True positive rate (TPR) was plotted in the function of the false positive rate (FPR) for meningococcal serogroup A (**a**), C (**b**), W (**c**), and Y (**d**). The estimated threshold, which provided a specificity of ≥90% are shown as vertical dotted lines

A significant AUC > 0.80 was observed for MenC, MenW and MenY (Fig. 3b-d). An AUC of the ROC of 0.69 (95% CI 0.65–0.74, *p*-value < 0.001; Fig. 3a) was observed for MenA, and the salivary test was therefore not considered adequate.

Based on the pre-specified criterium of a specificity of ≥90%, a threshold of MenC-PS specific IgG in saliva of 3.54 ng/mL was estimated (Table 4). Using this threshold, a TPR, PPV and NPV of 0.86 (740/863), 0.95 (740/777), and 0.74 (348/471) was calculated, respectively. Compared to MenC, lower salivary IgG thresholds were observed for MenW and MenY (2.00 and 1.82 ng/mL, respectively). For MenW, the calculated TPR, PPV and NPV were 0.62 (258/419), 0.94 (258/275), and 0.50 (164/325), respectively (Table 4) and for MenY, the TPR, PPV and NPV were 0.51 (235/458), 0.95 (235/2548), and 0.36 (126/349), respectively.

**Table 4:** Estimated threshold for salivary IgG, true positive rate (TPR), false positive rate (FPR), positive predictive value (PPV) and negative predictive value (NPV) with ROC curve analysis with a functional antibody titer (rSBA) >=128 as correlate of protection

Serogroup	Threshold (ng/mL)	TPR	FPR	PPV	NPV
MenC	3.54	0.86	0.10	0.95	0.74
MenW	2.00	0.62	0.09	0.94	0.50
MenY	1.82	0.51	0.09	0.95	0.36

For MenC, MenW and MenY, all (100%) samples collected 1 month and 1 year after the (booster) meningococcal vaccination with a saliva concentration above the estimated threshold showed sufficient protective serum rSBA titers for the corresponding serogroup (rSBA titer ≥128). Of all samples collected before the vaccination, 37/416 (8.9%), 17/200 (8.5%) and 13/199 (6.5%) with a saliva concentration above the estimated threshold had rSBA titers < 128 for MenC, MenW and MenY, respectively.

## Discussion

In this study, we showed that salivary IgG levels significantly correlated with bactericidal antibody titers in serum after both primary (MenA, MenW and MenY) and booster (MenC) vaccination. The best ROC curve (AUC of 0.95) was obtained after booster vaccination with MenC conjugate vaccine resulting in an estimated minimum threshold of MenC-PS specific salivary IgG ≥3.54 ng/mL as surrogate of protection in saliva. An adequate AUC (> 0.80) was also observed for MenW and MenY, suggesting that the saliva test may provide a screening tool to evaluate protection up to one year after primary vaccination. The minimum threshold in saliva as surrogate of protection was estimated to be 2.00 and 1.82 ng/mL for MenW- and MenY-PS specific IgG, respectively. MenA rSBA titers in serum correlated weakly with MenA-PS specific IgG levels in serum resulting in a less fitting ROC curve with a low AUC (0.69), suggesting that the saliva test is inaccurate as surrogate of protection for MenA after primary vaccination.

Saliva testing has important (cost-effective) advantages, particularly in studies that assess the impact of large-scale vaccination campaigns or in populations where blood sampling is difficult (ie. young children or resource poor settings) [21]. Despite this advantage and the growing interest in saliva as a tool to measure antibody levels in vaccine studies, no studies have evaluated the relationship between meningococcal salivary antibody levels and clinical protection against disease. Due to the absence of complement in saliva, salivary antibodies are not able to kill the meningococcus, but may have an important role in prevention of attachment of meningococci to epithelial cells [22–24]. Previous studies indicated that saliva IgG upon meningococcal conjugate vaccination against serogroup A, C, W and Y, rather than IgA, may be used for vaccine response monitoring because salivary IgG appears mostly serum-derived [12–14, 25] and thus reflects serum IgG levels upon vaccination that correlate with serum rSBA titers. Previously, we showed a strong correlation between IgG in serum and IgG in saliva for MenC, MenW and MenY, and a lower correlation for MenA [26]. In the current study, we confirmed good correlations between serum IgG levels and serum rSBA titers for meningococcal serogroup C, W and Y, in particular one month and one year after the (booster) vaccination. Therefore, salivary IgG levels might be used as surrogate marker of serum IgG antibody levels in serum. Taken together, this seems to indicate that it is possible to define a salivary antibody threshold that represents the protective status (rSBA≥128) after meningococcal vaccination.

After a single primary vaccination for MenA, MenW and MenY, we found a lower correlation between salivary IgG levels and rSBA titers as compared to a booster vaccination for MenC. There are several factors that might contribute to this lower correlation after primary vaccination. First, the ratio of total immunoglobulins relative to the IgG (Ig/IgG) that enhances complement mediated-killing may be higher after booster vaccination than primary vaccination. This may indicate that after primary vaccination also other Ig (ie. IgA or IgM) might enhance complement-mediated killing. Second, it could be possible that previous colonization has induced salivary IgG antibodies in some individuals leading to a weaker correlation with serum IgG levels and as a consequence overestimation of protection in case of a saliva test [27, 28]. Indeed, we observed false positive saliva samples at baseline for all serogroups (samples with a saliva concentration above the estimated threshold but with an rSBA titer < 128), indicating that the saliva test is less accurate as surrogate in unvaccinated populations or not recently immunized individuals. Third, the presence of antibodies against outer-membrane proteins, which are not measured by the PS-specific saliva test, in contrast to the SBA which includes a broader spectrum of functional antibodies, might lead to an underestimation of protection via the saliva test (false negative). All three factors could explain the lower correlation after primary vaccination, which results then in a lower performance of the saliva test as surrogate of protection. For MenA, the saliva test appeared not to be accurate enough (AUC < 0.80). As previously described for MenA, cross-reactive antibodies might explain the weak correlation between salivary IgG levels and serum rSBA titers [28]. These cross-reactive antibodies probably bind to the PS in the saliva test, but are not functional and therefore not contributing in the SBA. Nevertheless, the observed high AUC for MenC, MenW and MenY seems to provide an accurate threshold to discriminate between unprotected and protected individuals (ie. rSBA titers of < 128 and ≥ 128, respectively), especially after (booster) vaccination.

Our results showed that all samples that tested positive in the saliva test were indeed also positive in the SBA (true positive), indicating that the saliva test provides indeed an adequate tool to monitor vaccine responses up to one year after both primary and booster meningococcal vaccination. The use of the saliva test to discriminate between protection or not would have resulted in a reduction of the amount of elaborate serum SBAs in this meningococcal vaccine study by 87, 63 and 57% for MenC, MenW and MenY, respectively. Next to the advantages of taking saliva samples instead of blood, saliva IgG testing is easier than the SBA, less time-consuming and therefore more cost-effective. However, it has to be noted that some samples collected one month or one year after the (booster) vaccination though positive in the SBA were negative in the saliva test (false negative) ranging from 14% for MenC to 49% for MenY. The salivary threshold estimated in the current study should therefore be used as minimal threshold for surrogate of protection. Individuals with a salivary IgG levels above the estimated threshold will be protected but it is advisable to evaluate also serum SBA titers for all samples with a salivary IgG level below this minimal threshold.

A limitation of this study was that only samples of children aged 10–15 years were used here to identify the salivary thresholds. Therefore, these salivary thresholds as surrogate of protection have to be validated in other large meningococcal vaccine trials, preferably studies that include participants with a wide age range. In addition, samples were collected only up to one year after vaccination. Whether saliva samples can be used as a surrogate of protection in the long term after vaccination has to be investigated as well.

## Conclusions

In conclusion, the saliva test offers an alternative tool to screen vaccine responses up to one year after meningococcal vaccination against serogroups C, W and Y, with a high specificity and good sensitivity in particular after booster vaccination. Before the saliva test can be used as surrogate of protection for clinical protection against IMD, it is required to validate the proposed thresholds of the current study in future (large) longitudinal clinical meningococcal vaccination studies.

## Abbreviations

AUC: Area under the curve
IMD: Invasive meningococcal disease
MCVs: Meningococcal polysaccharide conjugate vaccines
MenA: Meningococcal serogroup A
MenACWY-TT: Tetanus toxoid conjugate quadrivalent MenACWY vaccine
MenC: Meningococcal serogroup C
MenC-TT: Tetanus toxoid conjugated monovalent MenC vaccine
MenW: Meningococcal serogroup W
MenY: Meningococcal serogroup Y
NPV: Negative predictive value
PPV: Positive predictive value
PS: Polysaccharide
ROC: Receiver operating characteristic
rSBA: Serum bactericidal assay using rabbit complement
TPR: True positive rate
